# Evaluation of PCR Based Assays for the Improvement of Proportion Estimation of Bacterial and Viral Pathogens in Diarrheal Surveillance

**DOI:** 10.3389/fmicb.2016.00386

**Published:** 2016-03-30

**Authors:** Hongxia Guan, Jingyun Zhang, Yong Xiao, Dan Sha, Xia Ling, Biao Kan

**Affiliations:** ^1^Wuxi Center for Disease Control and PreventionWuxi, China; ^2^State Key Laboratory for Infectious Disease Prevention and Control, National Institute for Communicable Disease Control and Prevention, Chinese Center for Disease Control and PreventionBeijing, China; ^3^Collaborative Innovation Center for Diagnosis and Treatment of Infectious DiseasesHangzhou, China

**Keywords:** diarrheal disease, diarrheogenic pathogen, PCR, bacterial pathogens, viral pathogens

## Abstract

Diarrhea can be caused by a variety of bacterial, viral and parasitic organisms. Laboratory diagnosis is essential in the pathogen-specific burden assessment. In the pathogen spectrum monitoring in the diarrheal surveillance, culture methods are commonly used for the bacterial pathogens' detection whereas nucleic acid based amplification, the non-cultural methods are used for the viral pathogens. Different methodology may cause the inaccurate pathogen spectrum for the bacterial pathogens because of their different culture abilities with the different media, and for the comparison of bacterial vs. viral pathogens. The application of nucleic acid-based methods in the detection of viral and bacterial pathogens will likely increase the number of confirmed positive diagnoses, and will be comparable since all pathogens will be detected based on the same nucleic acid extracts from the same sample. In this study, bacterial pathogens, including diarrheagenic *Escherichia coli* (DEC), *Salmonella* spp., *Shigella* spp., *Vibrio parahaemolyticus* and *V. cholerae*, were detected in 334 diarrheal samples by PCR-based methods using nucleic acid extracted from stool samples and associated enrichment cultures. A protocol was established to facilitate the consistent identification of bacterial pathogens in diarrheal patients. Five common enteric viruses were also detected by RT-PCR, including rotavirus, sapovirus, norovirus (I and II), human astrovirus, and enteric adenovirus. Higher positive rates were found for the bacterial pathogens, showing the lower proportion estimation if only using culture methods. This application will improve the quality of bacterial diarrheagenic pathogen survey, providing more accurate information pertaining to the pathogen spectrum associated with finding of food safety problems and disease burden evaluation.

## Introduction

Diarrhea is usually a symptom associated with infections of the intestinal tract, and can be caused by a variety of bacterial, viral, and parasitic organisms. Diarrhea occurs worldwide with wide-ranging characteristics and seasonal distributions. Globally, there are nearly 1.7 billion cases of diarrheal disease each year. This illness poses a significant global public health problem (Fischer Walker et al., 2010), and is a leading cause of child mortality and morbidity. The spread and prevalence of diarrheal diseases is closely related with regional, social, and economic development, along with geographical living habits (Bier et al., 2015; Letchumanan et al., 2015). Accurate identification of diarrheal etiologic agents not only results in improved treatment regimens, but also plays an important role in the analysis of risk factors associated with diarrheal prevalence. Successful pathogenic identification also facilitates location of the pathogenic source, improvements in food processing and supply management, and food safety promotion.

A number of studies have been performed in recent years to facilitate the determination of the pathogenic spectrum of diarrheal diseases. Although the results varied, the most common pathogens identified as causative agents of diarrheal disease were rotavirus, norovirus, diarrheagenic *Escherichia coli* (DEC), *Salmonella* spp., *Shigella* spp. and *Campylobacter* (DuPont, 2009; Scallan et al., 2011; Kotloff et al., 2013; Yu et al., 2015). In China, enteric viruses (predominantly norovirus and rotavirus) resulted in a higher number of diarrheal disease cases than bacterial causative agents (Liu and Zhang, 2013). However, other reports suggested that enteric bacteria such as DEC, *Salmonella* spp. and *Shigella* spp. were the dominant pathogenic agents (Zhu et al., 2013) associated with diarrhea (Qu et al., 2012; Zhu et al., 2013). *Vibrio parahaemolyticus* was more commonly observed in coastal regions (Li et al., 2014; Zhang et al., 2014).

The etiology of diarrhea can be difficult to determine using clinical symptoms alone, and laboratory diagnosis is often important. Diarrheogenic viruses are predominantly detected using molecular biological methods. For bacterial diarrhea, the isolation of pathogenic strains using bacteriological culturing techniques is still considered the “gold standard.” However, detection results in the assays can be influenced by many factors, such as bacterial load in the specimens, the experience of the laboratory technicians, and the cultural abilities for the different media and protocols. Additionally, treatment with antibiotics will greatly affect the isolation, leading to false negative results. The differences in the detection sensitivities associated with culture and nucleic acid-based methods might cause the lower estimation of bacterial pathogens compared to the viral pathogens in the same sample collections. To ameliorate problems related to the clinical treatment and epidemiological surveys associated with diarrheal pathogens, efforts have been made to reduce the turnaround times and improve the success rates associated with detection methods. These efforts have included the development of appropriate PCR-based detection assays (Amar et al., 2007; Harrington and Buchan, 2015).

Molecular detection methods are generally more sensitive than culture methods. Sample screening by molecular methods before strain isolation was supposed to be more efficient than isolating strains directly from all samples, because in the former strategy, more attention will be put on the samples positive in screening detection, the labor cost of detection will be reduced, and the positive detection rates will be increased. For bacterial pathogens needing an enrichment step before isolation, such as *Salmonella* spp. and *Vibrio* spp., molecular detection on stool nucleic acids might miss some samples with low bacterial loads, and more infectious samples will be identified by detecting the nucleic acids of enriched cultures. In this study, we compared the positive detection rates of culture methods, PCR-based detection on nucleic acids of fecal samples, and PCR-based detection on nucleic acids of enriched cultures. The application of PCR-based assays in the screening of *Salmonella* spp., *Shigella* spp., DEC, and *Vibrio* spp. was evaluated. Five common diarrheal viruses (rotavirus, sapovirus, norovirus, human astrovirus, and enteric adenovirus) were detected, and the composition of the pathogenic spectrum associated with 334 diarrheal samples was analyzed. Accordingly, an efficient detection process based on nucleic acid testing was established. The developed method can be applied to the surveillance of infectious diarrhea, leading to a rapid and accurate diagnosis, facilitating effective monitoring of diarrheal disease in clinical laboratories.

## Materials and methods

### Sample collection

A total of 334 fecal diarrheal samples were obtained from six sentinel hospitals from April to December, 2013. From April to December, 2013, stool specimens were collected from 334 outpatient diarrheal cases. The patients from whom the samples were collected were aged from 6 months to 89 years old. Thirty-two of the patients (9.6%) were under 5 years of age. The sample numbers for each month were 11, 16, 45, 71, 37, 42, 43, 53, and 16 (April to December), respectively.

**Figure 1:**
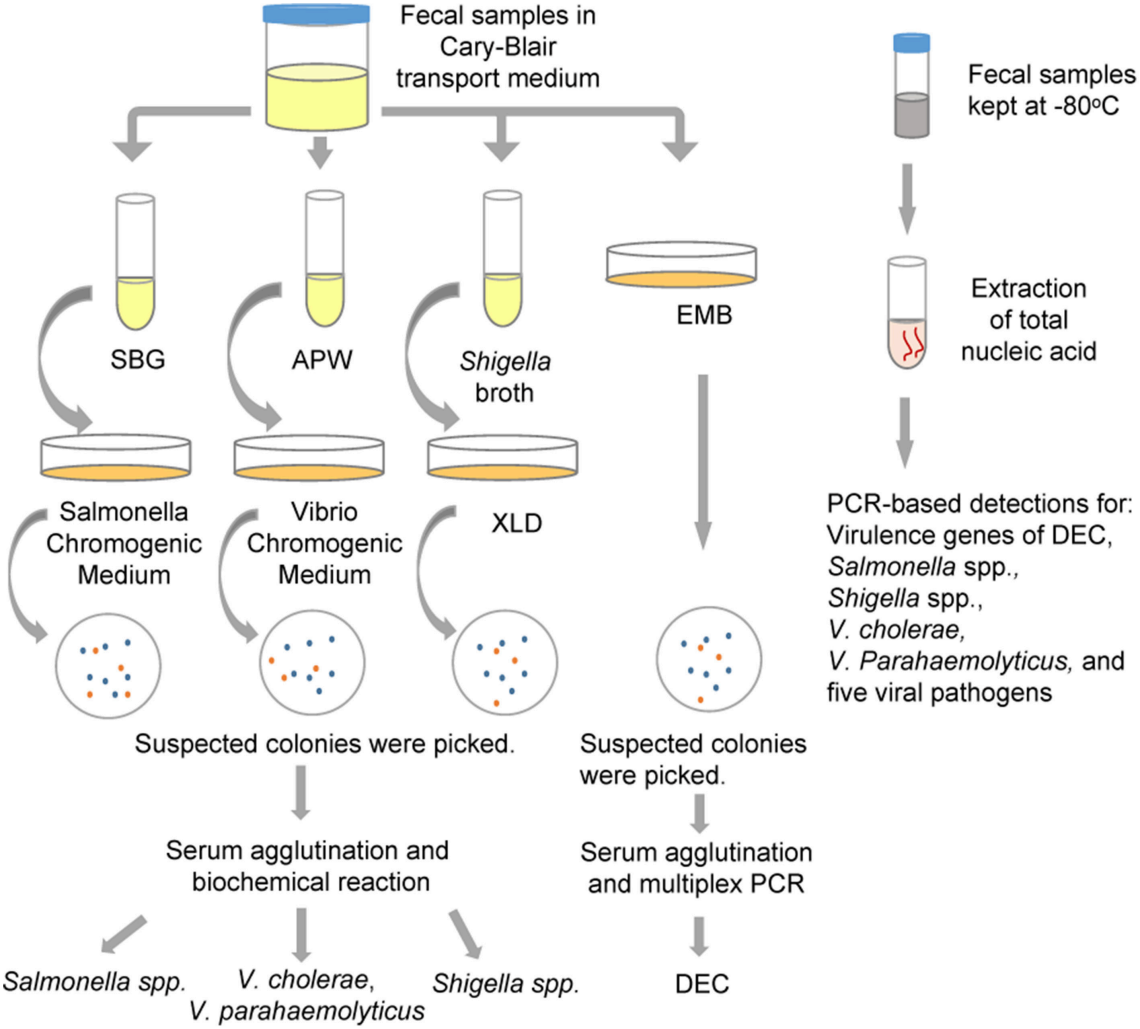
**The protocol for multiple pathogen detection used in the study**. APW, Alkaline Peptone Water; DEC, diarrheagenic *Escherichia coli*; EMB, Eosin Methylene Blue agar; SBG, Selenite Brilliant Green Broth; XLD, Xylose Lysine Deoxycholate medium.

Each fecal sample was divided into two parts: one was used for the detection of bacterial pathogens, and was preserved in Cary-Blair transport medium and stored at 4°C until tested (within 24 h); the other part was used for the detection of viral pathogens, and was collected in a 20-ml centrifugal tube and kept at −80°C until further processed. The flow chart of detection in this study was shown in Figure 1.

Diarrhea was defined as the daily excretion of three or more unformed stools, including watery stools, mucous and purulent blood stools, where the patients had not taken antibiotics.

### Conventional culture methods

As shown in Figure 1, all fecal specimens were detected using the routine culture methods for the isolation of *Salmonella* spp., *Shigella* spp., *V. cholerae, V. parahaemolyticus* and five types of DEC strains [enteroadhesive *E. coli* (EAEC), enterohemorrhagic *E. coli* (EHEC), enteroinvasive *E. coli* (EIEC), enteropathogenic *E. coli* (EPEC), and entertoxigenic *E. coli* (ETEC)]. Fecal samples suspended in Cary-Blair medium were inoculated in Selenite Brilliant Green Broth (SBG) and Alkaline Peptone Water (APW) to enrich strains of *Salmonella* spp. and *Vibrio* spp., respectively. After being incubated overnight at 37°C, the SBG cultures were sub-cultured on Salmonella Chromogenic Medium, and purple colonies were picked as suspected *Salmonella*. The APW cultures were sub-cultured on Vibrio Chromogenic Medium, and mauve and turquoise-blue colonies were picked as suspected *V. parahaemolyticus* and *V. cholerae* respectively. These suspected colonies were examined using serum agglutination tests and identification was confirmed by systematic biochemical reaction with VITEK 2 Gram-Negative Identification (GN) cards (BioMerieux, France). Simultaneously, fecal samples were streaked on Xylose Lysine Deoxycholate medium (XLD) and Eosin Methylene Blue agar (EMB) to identify *Shigella* spp. and DEC, respectively. Suspected *Shigella* spp. colonies (pink, translucence without black centers on XLD plates) were identified using serum agglutination and VITEK. For the suspected DEC colonies (purple with metallic shine on EMB agar), the strains that resulted in positive serum agglutination tests were identified by multiplex PCR targeting virulence genes. One hundred and twenty two stool samples collected from August to October, 2013, were inoculated in *Shigella* broth, and incubated overnight at 42°C using anaerobic growth conditions. The broth cultures were subsequently sub-cultured onto XLD plates to facilitate isolation of *Shigella* spp. All media were obtained from Luqiao Co., Beijing, China.

### Total nucleic acid extraction from the stool samples

Stool samples stored at −80°C were thawed within a month prior to processing. A 200 μl aliquot of the thawed material was transferred to a sample cartridge for the total nucleic acid extraction using the MagNA Pure LC Total Nucleic Acid Isolation Kit on the MagNA Pure LC 2.0 instrument (Roche Diagnostics, Indianapolis, IN). For non-liquid stool samples, 1 ml of 0.9% sodium chloride solution was added to each sample, the mixture was thoroughly mixed using a vortex and allowed to settle for 2 min. The supernatant (200 μl) was then used for the total nucleic acid extraction. For each stool sample, nucleic acid was also extracted using the MagNA Pure LC Total Nucleic Acid Isolation Kit using 200 μl aliquots of SBG and APW culture broths. Nucleic acid was also extracted from bacterial lawns associated with the initial streak areas of EMB plates following suspension in distilled water. The nucleic acid were eluted with 50 μl of elution buffer and stored at −80°C until further processed.

### PCR tests

Virulence genes of DEC strains were screened using a multiplex PCR assay with TaKaRa Ex Taq polymerase (TaKaRa, China) and the primers listed in Table 1 (Zhao et al., 2011). The detection targets included *eaeA* of EPEC, *stx1* and *stx2* of EHEC, *lt* and *stIb* of ETEC, *aggR* of EAEC, and *ipaH* of EIEC & *Shigella.* The following PCR cycling conditions were used: 94°C for 5 min, 30 cycles of 94°C for 60 s, 57°C for 60 s, and 72°C for 60 s, with a final extension of 72°C for 10 min. The nucleic acid extracted from fecal samples and EMB cultures were used as PCR templates, respectively. The amplification products were analyzed using the QIAxcel capillary electrophoresis instrument and the QX DNA Screening Cartridge (Qiagen, Hilden, Germany).

**Table 1:** **Primer sequences for multiplex PCR of DEC (Zhao et al., 2011)**.

**Targets**	**Sequence (5′−3′)**	**Product length (bp)**	**Final concentration (nM)**
*rrs*	F: CCCCCTGGACGAAGACTGAC; R: ACCGCTGGCAACAAAGGATA	401	100
*ipaH*	F: GTTCCTTGACCGCCTTTCCGATACCGTC; R: GCCGGTCAGCCACCCTCTGAGAGTAC	619	200
*stx1*	F: AAATCGCCATTCGTTGACTACTTCT; R: TGCCATTCTGGCAACTCGCGATGCA	370	400
*stx2*	F: CAGTCGTCACTCACTGGTTTCATCAC; R: GGATATTCTCCCCACTCTGACACC	283	400
*eaeA*	F: TCAATGCAGTTCCGTTATCAGTT; R: GTAAAGTCCGTTACCCCAACCTG	482	400
*aggR*	F: GTATACACAAAAGAAGGAAGC; R: ACAGAATCGTCAGCATCAGC	254	600
*lt*	F: TCTCTATGTGCATACGGAGC; R: CCATACTGATTGCCGCAA	322	500
*stIb*	F: TGCTAAACCAGTAGAGTCTTCAAAA; R: GCAGGATTACAACACAATTCACAGCAG	138	400

*Salmonella* spp. and *Shigella* spp. were simultaneously detected from stool samples, SBG broths and *Shigella* broths, respectively, using a *Salmonella*/*Shigella* duplex real-time PCR kit (Huirui, Shanghai, China). *V. cholerae* and *V. parahaemolyticus* were detected from stool samples and APW broths using a *V. cholerae*/*V. parahaemolyticus* duplex real-time PCR kit (Huirui, Shanghai, China).

Five viral pathogens were detected from stool nucleic acid using singleplex real-time PCR assays with a QuantiTect Probe RT-PCR Kit (QIAGEN, Hilden, Germany). The primers and probes (Table 2) were recommended by Jiangsu Center for Disease control and Prevention for the surveillance of diarrheal diseases.

**Table 2:** **Sequences of primers and probes used in this study**.

**Pathogens or targets**	**Primers and probes**	**Sequences (5′−3′)**	**Product lengths**
Rotavirus	NVP3_F	ACCATCTWCACRTRACCCTC	/
	NVP3_R	GGTCACATAACGCCCC	
	NVP3_R1	GGTCACATAACGCCCCTATA	
	NVP3_Probe	FAM-ATGAGCACAATAGTTAAAAGCTAACACTGTCAA-TAMARA	
Sapovirus	SV_F	GCTGTTSCYACTGGTGCA	/
	SV_R	GGCATCCTGTCRTTCCAAGCA	
	SV_Probe	FAM-CCAATCSAATGTCCCTGAGGCAATACGSAA-TAMARA	
Norovirus I	COG1_F	CGYTGGATGCGNTTYCATGA	/
	COG1_R	CTTAGACGCCATCATCATTYAC	
	RING1(a)_TP	FAM-AGATYGCGATCYCCTGTCCA-TAMARA	
	RING1(b)_TP	FAM-AGATCGCGGTCTCCTGTCCA-TAMARA	
Norovirus II	COG2_F	CARGARBCNATGTTYAGRTGGATGAG	/
	COG2_R	TCGACGCCATCTTCATTCACA	
	RING2_TP	FAM-TGGGAGGGCGATCGCAATCT-TAMARA	
Human astrovirus	AV1 AV2 Av_Probe	CCG AGT AGG ATC GAG GGT GCTTCTGATTAAATCAATTTTAA FAM-CTTTTCTGTCTCTGTTTAGATTATTTTAATCACC-TAMARA	/
Enteric adenovirus	Adeno.fwd	GCCACGGTGGGGTTTCTAAACTT	/
	Adeno.rev	GCCCCAGTGGTCTTACATGCACATC	
	Adeno.probe1	FAM-TGCACCAGACCCGGGCTCAGTACTCCGA-TAMARA	
*Salmonella* spp.	OMPCF	ATCGCTGACTTATGCAATCG	204 (Kwang et al., 1996)
	OMPCR	CGGGTTGCGTTATAGGTCTG	
*Shigella* spp.	virA-F	CTGCATTCTGGCAATCTCTTCACA	215 (Fukushima et al., 2003)
	virA-R	TGATGAGCTAACTTCGTAAGCCCTCC	
Internal control	IAC-f	CTAACCTTCGTGATGAGCAATCG	145 (Deer et al., 2010)
	IAC-r	GATCAGCTACGTGAGGTCCTAC	

The existence of PCR inhibitors in fecal nucleic acids was tested by the amplification of an internal control as introduced in reference (Deer et al., 2010) with modifications: the 200 bp DNA fragment (GenBank no. FJ357008) was synthesized in TaKaRa Dalian co. and ligated into vector pMD18-T, generating the internal control plasmid pMD18T-IAC. About 2 × 10^5^ copies of pMD18T-IAC, 200 nmol/l primers IAC-f and IAC-r (Table 2), 2 μl of fecal nucleic acids were added in a PCR reaction mixture, and amplified under the following conditions: 94°C for 5 min, 40 cycles of 94°C for 30 s, 55°C for 30 s, and 72°C for 30 s. A 145-bp fragment would be generated if no PCR inhibitors existed.

### Validation tests

For *Salmonella* spp. and *Shigella* spp., discrepant results between routine isolation and commercial real-time PCR kits were resolved following singleplex PCR targeting *ompC* in *Salmonella* spp. and *virA* in *Shigella* spp. (primers listed in Table 2). The amplified products were further sequenced using the PCR primers. To confirm the existence of DEC that were initially identified following multiplex PCR, amplified products were sequenced with the corresponding primers used in the multiplex assay.

### Statistical analysis

Statistical analysis of experimental data was performed using the chi-square test with SAS software version 9.2 (SAS Institute Inc., Cary, NC, USA). The two-tailed *p* value of < 0.05 indicated statistical significance.

## Results

### Bacterial pathogens isolated by culture method

Following routine bacterial culture methods, 18 *Salmonella* spp., two *Shigella* spp., six DEC, two *V. parahaemolyticus* and two *V. cholerae* were isolated. Among the 18 *Salmonella* strains, one was isolated from a child < 5 years of age with a positive detection rate of 3.8% (1/26); 17 were isolated from patients >5 years of age, with a positive detection rate of 5.5% (17/308). There was no significant difference between the positive detection rates associated with *Salmonella* spp. in the two age groups. All other pathogens were isolated from individuals >5 years of age.

The 18 *Salmonella* strains were distributed into nine serotypes, among which *S.* Enteritidis and *S.* Typhimurium were the two main serotypes. The two isolated *Shigella* strains were *S. flexneri* and *S. Sonnei*, respectively. The six isolated DEC strains were typed into five serogroups: O148 (two strains), O15, O153, O159, and O25. Both *V. parahaemolyticus* strains were categorized as O3:K6, and both *V. cholerae* strains were non-O1/non-O139.

### PCR detection of the bacterial pathogens in the specimens

The existence of bacterial pathogens in stool samples and enriched samples was detected using PCR-based methods. Validation of these results was performed by sequencing. The enriched samples used as part of this analysis included SBG cultures for *Salmonella* spp., *Shigella* broths for *Shigella* spp., APW cultures for *Vibrio* spp., and bacterial lawns grown on EMB agar for DEC. As shown in Table 3, 18 stool and 21 enriched samples were positive for DEC, eight stool and four enriched samples were positive for *Shigella* spp., two stool and two enriched samples were positive for *V. parahaemolyticus*, one stool and two enriched samples were positive for *V. cholerae*, and one stool and 22 enriched samples were positive for *Salmonella* spp. The PCR results of samples that were positive for *Shigella* or *Salmonella* were confirmed by sequencing of the amplified products. Conversely, five of the 18 amplified products generated by the multiplex PCR with fecal nucleic acids resulted in non-DEC sequences when sequencing reactions were performed. Of the 334 stool samples, 219 were examined for PCR inhibitors by the amplification of an internal control fragment carried on plasmid pMD18T-IAC. No PCR inhibitor was observed in the 219 samples, including the 22 samples with SBG broths positive for *Salmonella.*

**Table 3:** **Summary of the detection results in this study**.

**Pathogens**	**Culture**	**PCR-based methods**			
		**PCR of stool samples**		**PCR of enriched samples**	
		**+**	**Confirmed +**	**+**	**Confirmed +**
*Salmonella* spp.	18	1	1	22	22
*Shigella* spp.	2	8	8	4^a^	4
DEC	6	18	13	21	21
*V. parahaemolyticus*	2	2	ND^b^	2	ND
*V. cholerae*	2	1	ND	2	ND
Total	30	30		51	

a *Positive results from Shigella broth, 122 specimens from August to October*.

b *Not detected*.

The PCR-based detection results were compared with those generated following routine culture methods. For *Salmonella* spp., all culture-positive samples gave positive PCR results when SBG cultures were used as amplification templates. Additionally, PCR results showed an even greater number of positive samples than the initial culture results suggested. Similar observations were made for DEC and *Shigella* spp. However, most of the positive results generated following PCR analysis were obtained using EMB lawns for DEC and stool nucleic acid for *Shigella* spp. Following statistical analysis, a greater number of positive results were generated following PCR amplification using SBG for *Salmonella* spp., EMB cultures for DEC, and raw samples for *Shigella* spp. when compared to traditional culture and isolation methods. The observed differences were significant (*P* < 0.05) (Table 4).

**Table 4:** **Comparison of the detection results between PCR-based methods and culture/isolation method**.

**Culture**		**PCR-based methods**			
		**PCR of stool samples**		**PCR of enriched samples**	
		**+**	**−**	**+**	**−**
*Salmonella* spp.	+	1	17	18	0
	−	0	316	4	312
	*P*	< 0.0001		0.0455	
*Shigella* spp.	+	2	0	1	0
	−	6	326	3	118
	*P*	0.0143		0.0838	
DEC	+	2	4	5	1
	−	11	317	16	312
	*P*	0.0707		0.0003	

The results generated following the analysis of *V. parahaemolyticus* and *V. cholerae* were relatively consistent. Results observed following PCR amplification from fecal and enriched samples, were consistent with culture methods except where one sample showed a false negative result when fecal nucleic acid were used as the PCR template.

We estimated the sensitivities of the different methods for the target pathogens (Table 5). The detection of *Salmonella* spp. and DEC using the PCR method following enrichment showed the highest sensitivity. This sensitivity value was significantly higher than that associated with direct PCR detection from fecal samples. For *Shigella* spp., direct PCR amplification from fecal samples resulted in the highest sensitivity. The detection sensitivities associated with *Vibrio* spp. were not compared since the associated positive detection rates were relatively low.

**Table 5:** **Positive sample numbers and sensitivities of different methods for different pathogens**.

	**Positive number (sensitivity)**			
**Pathogen**	**PCR on stool nucleic acid**	**PCR on enriched samples**	**culture**	**Total positive number**
*Salmonella* spp.	1 (4.5%)	22 (100%)	18 (81.8%)	22
*Shigella* spp.	8 (100%)	4^a^ (100.0%)	2 (25.0%)	8
DEC	13 (46.4%)	21 (75%)	6 (21.4%)	28
*V. parahaemolyticus*	2 (100%)	2 (100%)	2 (100%)	2
*V. cholerae*	1 (50.0%)	2 (100%)	2 (100%)	2

a *Positive results from Shigella broth, 122 specimens from August to October*.

### The etiological spectrum of the most common viral targets using PCR

**Figure 2:**
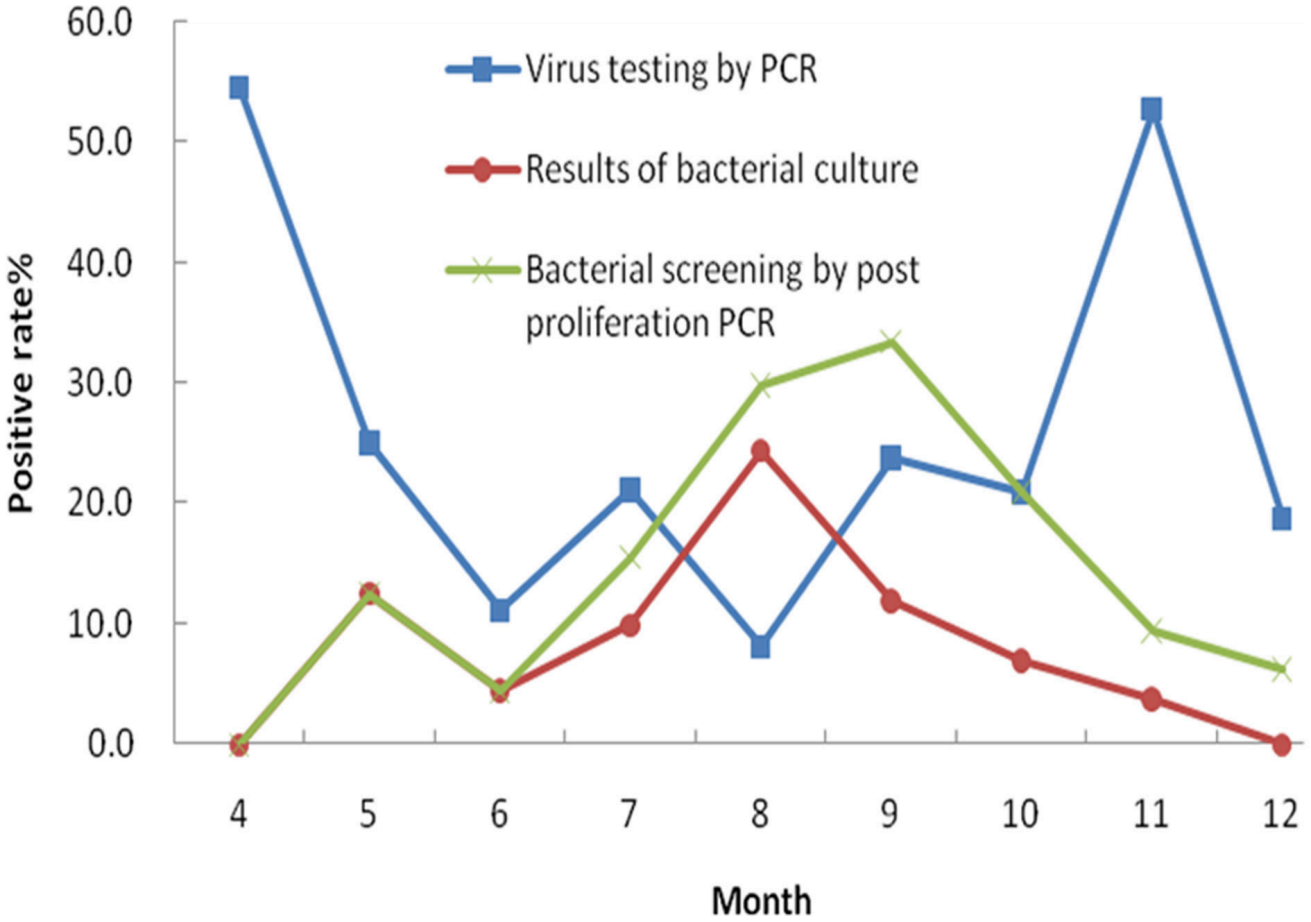
**Seasonal epidemic occurrence curve for viruses and bacteria**.

In this study, five common enteric viruses were detected in 334 fecal specimens using real time RT-PCR. Norovirus was the most common virus detected in the diarrheal samples (39 specimens, 11.7%). The second most common virus that was detected was rotavirus with a positive detection rate of 8.4% (28 specimens). Seven specimens tested positive for enteric adenovirus (2.1%), while human astrovirus and sapovirus were observed in five (1.5%) and four (1.2%) samples, respectively. A more detailed analysis of the patient profiles associated with these infections was performed. The results showed that 20.5% of the patients that were infected with norovirus were less than 5 years of age. This result was significantly different from the patient group containing individuals aged 5 years of age and older (*P* = 0.0136). A similar difference was also observed in the 28 patients who tested positive for rotavirus infection, with 25.0% of the infected cohort under 5 years of age (*P* = 0.0038). Additionally, seven samples were deemed co-infected by two different viruses following a combination of bacterial culture and PCR analysis. The most common pathogens found to be involved in co-infections included norovirus (6/13, 46.2%), DEC (5/13, 38.5%) and *Salmonella* spp. (4/13, 30.8%). We next compared seasonal distributions associated with bacterial and viral infections in the associated diarrheal cases. This was performed by combining the positively identified cases (bacterial and viral) that occurred each month (Figure 2). The curves show the seasonal characteristics associated with positive bacterial and viral identifications: higher positive detection rates occurred in cold seasons for viruses and higher positive detection rates occurred in the summer months for bacteria. In this study, we analyzed the difference in the detection rates of bacteria following both culture and PCR detection after enrichment. Higher detection rates were observed following nucleotide acid amplification than those observed following bacterial culture.

## Discussion

Diarrhea can be caused by numerous pathogens. Historically, surveillance methods used to detect diarrheal diseases have depended on the type of causative pathogen. Typically, bacterial pathogens are identified using culture and isolation methods, while viral pathogens are predominantly determined by nucleic acid-based methods. The culture and isolation of bacterial pathogens is labor-intensive and time-consuming with complicated procedures and low sensitivities (Chitkara, 2005; De Boer et al., 2010). For this reason, only relatively small numbers of diarrheal cases are examined for bacterial pathogens, especially in developing regions. Consequently, when diarrheal samples have been screened for both bacterial and viral pathogens, the prevalence of different pathogens and the pathogenic composition in these samples were often inaccurate considering the dramatic differences in the detection sensitivities of culture and nucleic acid-based methods. With this in mind, it is hoped that the increased exclusive application of nucleic acid-based methods in the detection of both bacterial and viral pathogens, will facilitate greater detection levels in relation to the causative agents, thereby improving the quality of diarrheal disease monitoring results, and improve the pathogen-specific disease burden assessment further. Many commercial detection kits are available, permitting the simultaneous detection of the most common diarrheagenic pathogens with high throughput and short detection times, such as BioFire FilmArray gastrointestinal panel and Luminex xTAG Gastrointestinal Pathogen Panel (Khare et al., 2014; Liu et al., 2014; Buss et al., 2015). However, up until recently these kits had not been routinely used in primary public healthcare laboratories due to high costs in the developing regions. In order to ameliorate the financial offset associated with the use of molecular detection methods, optimized protocols for high-efficiency low-cost pathogen detection methods are required.

**Figure 3:**
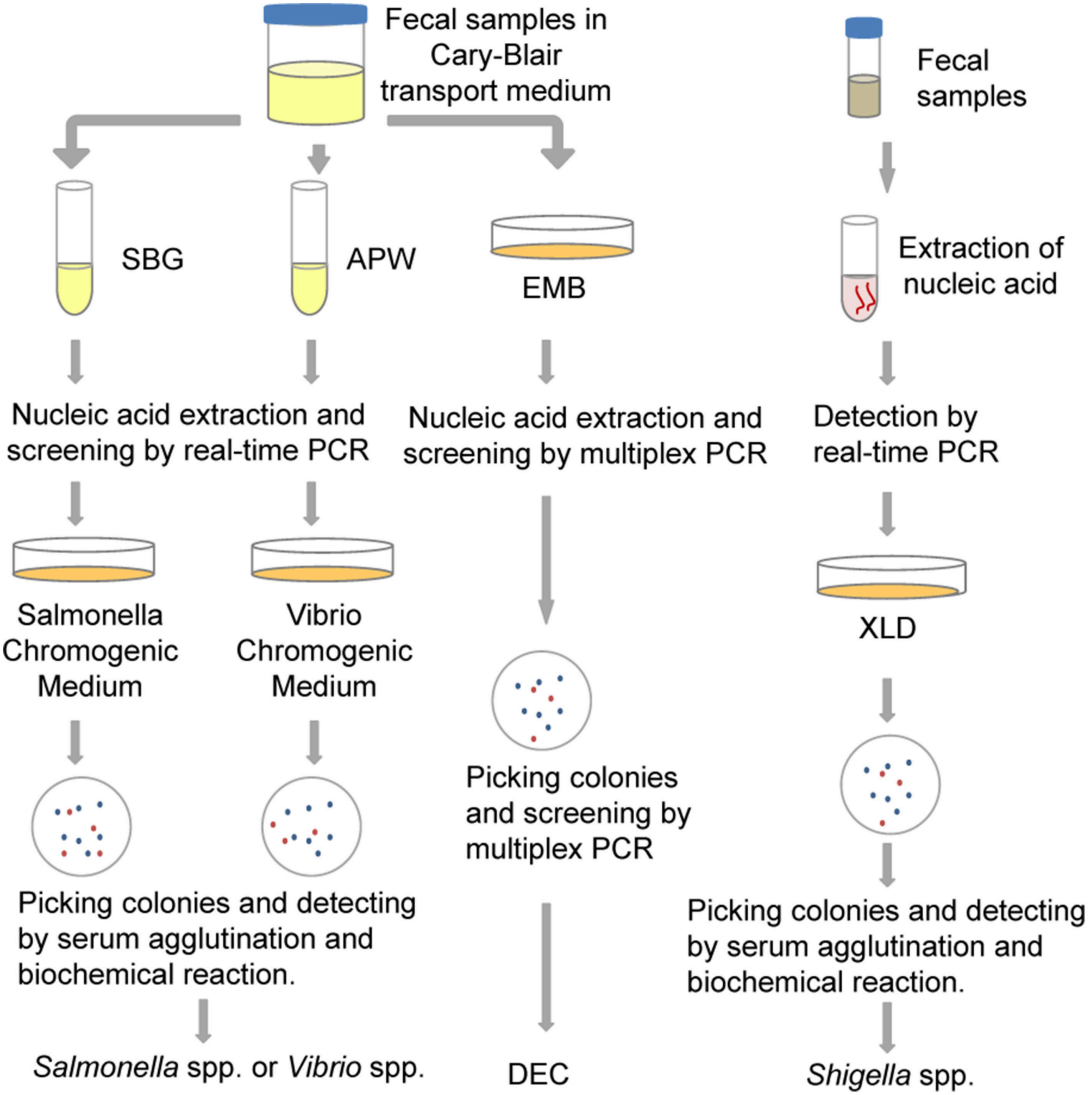
**The protocol for multiple pathogen surveillance (developed as a result of this study)**. APW, Alkaline Peptone Water; DEC, diarrheagenic *Escherichia coli*; EMB, Eosin Methylene Blue agar; SBG, Selenite Brilliant Green Broth; XLD, Xylose Lysine Deoxycholate medium.

In this study, we detected five common bacterial pathogens following analysis of 334 diarrheal samples using both culture and PCR-based methods. Total nucleic acid extracted from both raw stool samples and enriched cultures were used as templates for PCR-based assays. The detection efficiency associated with the different methods was compared. As a result of this study, a new protocol was estimated and established for the identification of these bacterial pathogens, as shown in Figure 3. It is hoped that these results will be helpful in the selection of appropriate detection methods and procedures that will improve the identification of diarrheal pathogens.

As previously reported (Dutta et al., 2001; Operario and Houpt, 2011; Buchan et al., 2013), nucleic acid-based detection systems used for bacterial pathogens result in higher detection rates than culture methods. The highest positive detection rate was observed when enriched cultures were examined, especially for *Salmonella* spp. and DEC. Nevertheless, in relation to the detection of bacterial pathogens, the effectiveness of nucleic acid-based methods varied depending on the pathogen type and enrichment method used.

When *Salmonella* spp. were analyzed, the highest positive detection rate (6.6%, 22/334) was observed when SBG cultures of fecal samples were detected using real-time PCR. Interestingly, the positive detection rate was only 0.3% (1/334) when the nucleic acid isolated from fecal samples were used for direct detection using the same real-time PCR assay. Previous reports have suggested that PCR inhibition factors exist in approximately 3–4% of extracted stool nucleic acid extracts (Iijima et al., 2004). We examined the stool nucleic acids of the 22 samples in the PCR reaction containing internal control plasmid pMD18T-IAC and primers IAC-f / IAC-r. The targeting 145-bp fragment was amplified in all 22 samples, and the existence of PCR inhibitors was excluded for the sample examined. We deduced that low bacterial load is one of the most common reasons for decreased positive detection rates when fecal sample nucleic acid are analyzed. By combining detection methods using real-time PCR with enrichment in SBG, the presence of *Salmonella* spp. in analyzed samples can be diagnosed within 8 h following receipt of the samples. This means that more time can be spent collecting colonies from positive samples, with the overall labor cost associated with selection and identification being markedly reduced.

*E. coli* strains are a major component of normal microflora in human. However, DEC strains are a common cause for diarrheal disease. Diagnosis of DEC using colony selection followed by biochemical identification and serum agglutination is time-consuming and laborious, and also results in many false positive and false negative results, due to the fact that pathogenic and non-pathogenic strains exhibit identical colonial morphologies. Additionally, there are various serotypes associated with the pathogenesis of *E. coli* strains (Croxen et al., 2013). In this study, five candidate colonies were selected from EMB plates and subjected to biochemical identification and serum agglutination. Approximately 1670 colonies from 334 specimens were screened. Only six DEC strains were observed following this analysis, resulting in a DEC-positive detection rate of 1.8%. In contrary, when using the PCR-based detection method, 13 stool samples and 21 enriched samples were confirmed as positive for DEC, showing that a large number of DEC strains were not detected using the colony selection method. Commercial real-time PCR kits commonly facilitate the detection of one or two targets per reaction. Because several DEC-specific virulence genes were detected in this study, a multiplex PCR assay, allowing for the simultaneous detection of five types of DEC, was explored as part of this study instead of real-time PCR assays. Following the sequencing confirmation of the amplicons, we found that some of the PCR amplicons were generated by non-specific amplification, resulting in false positives. Further false positives were observed when nucleic acid associated with the stool samples was examined. This might be caused by the complicated composition of microbial populations that colonize the human gastrointestinal tract. But following enrichment of the fecal samples by using EMB media, both the sensitivity and the specificity of the multiplex PCR assay were enhanced. As a result, the detection of DEC-virulence genes using multiplex PCR from enriched cultures on selective media such as EMB is feasible. Indeed, this procedure is more intuitive and less costly due to the fact that nucleic acid extraction from stool samples is not required.

*Shigella* strains were also detected using colony selection (from XLD plates) along with real-time PCR where nucleic acid extracted from stool samples were used as templates. The latter method resulted in a higher number of *Shigella* strains being identifed. During the early phases of this project, 122 diarrheal samples were incubated in *Shigella* broth and cultured under anaerobic conditions. When these samples were screened for *Shigella* spp. using real-time PCR, the same four samples gave positive results whether nucleic acid isolated from fecal samples or nucleic acid isolated from enriched cultures were examined. However, amplification of the nucleic acid derived from the enriched cultures resulted in lower Ct values. This implied that media-enrichment was an effective method for enriching the growth of *Shigella* strains.

Only four *Vibrio*-positive samples were identified in this study. As a result of the low detection rate, a statistical analysis was not performed on these results. The results generated from the culture-based method were in agreement with the real-time PCR method. However, the real-time PCR assay resulted in the generation of reduced Ct values when APW enriched cultures were used as amplification templates, for the reason that APW broth may facilitate the proliferation of *Vibrio*s spp.

In addition to bacterial pathogens, five viral pathogens were detected using real-time PCR or RT real-time PCR. The resultant pathogenic spectrum was analyzed in diarrheal outpatient inhabitants of Wuxi city. Sampling was performed over a nine-month period, including the summer and winter diarrheal seasons. The data generated reflected the situation associated with diarrheagenic pathogens in this area.

As a result of this analysis, we observed that the incidence of bacterial diarrhea is higher in summer months (from July to September) than for the rest months of the year in Wuxi city. *Salmonella* spp. and DEC represented the most common causes of diarrheal illness during this period. The overall incidence of viral infections is higher than that of bacterial infections. As previously reported (Verhoef et al., 2008; Dey et al., 2012), viral infections occurred throughout the year, peaking during the cold season. The two most common viral diarrheal pathogens were norovirus and rotavirus. These results were in agreement with recent surveillance data generated in Wuxi, but differed from results observed in Changchun and Chengdu of China where the predominant pathogen causing diarrheal illness was rotavirus (Xie et al., 2012; Zhang et al., 2012). In Henan province, DEC, rotavirus, *Shigella* spp., *Salmonella* spp., and norovirus were the five leading diarrheagenic pathogens in 2007 (Zhu et al., 2013). In Beijing, *Shigella* spp., *Vibrio* spp., *Salmonella* spp., and DEC were the four most common bacterial pathogens observed in diarrheal outpatients between April, 2010 and December, 2011 (Qu et al., 2012). In Shenzhen of China, a five-year surveillance study (2007 to 2012) showed that *V. parahaemolyticus* was the leading cause of diarrheal illness, followed by *Salmonella* spp., DEC and *Shigella* spp. (Li et al., 2014). It should be noted that all studies mentioned here facilitated the detection of bacterial pathogens using common bacterial culture methods, while our study utilized both culture and nucleic acid-based methods. Our results suggest that data acquired by culture method alone might lead to the underestimation of infection rates and proportions of pathogenic bacteria in the diarrheal patients. Using PCR-based detection methods (following bacterial enrichment), higher positive detection rates associated with bacterial pathogens were observed, and the data were much closer to the actual infection rates.

In summary, we estimated the PCR-based and cultural methods for the multiple bacterial pathogens survey of diarrheal cases. The data show that in the etiological spectrum survey especially when viral pathogens are included, it will be more reasonable to use the PCR-based method for the bacterial pathogen detection, instead of the cultural isolation. Enrichment of fecal sample before PCR detection is critical to increase the sensitivity. Many PCR-based protocols and commercial kits detect pathogen-specific targets directly in the fecal samples, therefore the positive rates of pathogens will be lower estimated. The protocol established in this study will prompt the identification of bacterial pathogens in diarrheal patients and improve the monitoring quality associated with bacterial diarrhea.

## Author contributions

HG and JZ contributed equally to this work, completed all experiments. YX and DS gave assistance for the research. XL and BK were responsible for management of the research.

### Conflict of interest statement

The authors declare that the research was conducted in the absence of any commercial or financial relationships that could be construed as a potential conflict of interest.

## References

[B1] Amar C. F. East C. L. Gray J. Iturriza-Gomara M. Maclure E. A. Mclauchlin J. (2007). Detection by PCR of eight groups of enteric pathogens in 4,627 faecal samples: re-examination of the English case-control Infectious Intestinal Disease Study (1993-1996). Eur. J. Clin. Microbiol. Infect. Dis. 26, 311–323. 10.1007/s10096-007-0290-8 17447091

[B2] Bier N. Schwartz K. Guerra B. Strauch E. (2015). Survey on antimicrobial resistance patterns in *Vibrio vulnificus* and *Vibrio cholerae* non-O1/non-O139 in Germany reveals carbapenemase-producing *Vibrio cholerae* in coastal waters. Front. Microbiol. 6:1179. 10.3389/fmicb.2015.01179 26579088 PMC4623411

[B3] Buchan B. W. Olson W. J. Pezewski M. Marcon M. J. Novicki T. Uphoff T. S.. (2013). Clinical evaluation of a real-time PCR assay for identification of *Salmonella, Shigella, Campylobacter* (*Campylobacter jejuni* and *C. coli*), and shiga toxin-producing *Escherichia coli* isolates in stool specimens. J. Clin. Microbiol. 51, 4001–4007. 10.1128/JCM.02056-13 24048539 PMC3838069

[B4] Buss S. N. Leber A. Chapin K. Fey P. D. Bankowski M. J. Jones M. K.. (2015). Multicenter evaluation of the BioFire FilmArray gastrointestinal panel for etiologic diagnosis of infectious gastroenteritis. J. Clin. Microbiol. 53, 915–925. 10.1128/JCM.02674-14 25588652 PMC4390666

[B5] Chitkara Y. K. (2005). Limited value of routine stool cultures in patients receiving antibiotic therapy. Am. J. Clin. Pathol. 123, 92–95. 10.1309/EQP21KEMBB6EHG9B 15762283

[B6] Croxen M. A. Law R. J. Scholz R. Keeney K. M. Wlodarska M. Finlay B. B. (2013). Recent advances in understanding enteric pathogenic *Escherichia coli*. Clin. Microbiol. Rev. 26, 822–880. 10.1128/CMR.00022-13 24092857 PMC3811233

[B7] De Boer R. F. Ott A. Kesztyüs B. Kooistra-Smid A. M. (2010). Improved detection of five major gastrointestinal pathogens by use of a molecular screening approach. J. Clin. Microbiol. 48, 4140–4146. 10.1128/JCM.01124-10 20861334 PMC3020836

[B8] Deer D. M. Lampel K. A. González-Escalona N. G. (2010). A versatile internal control for use as DNA in real-time PCR and as RNA in real-time reverse transcription PCR assays. Lett. Appl. Microbiol. 50, 366–372. 10.1111/j.1472-765X.2010.02804.x 20149084

[B9] Dey S. K. Phathammavong O. Nguyen T. D. Thongprachum A. Chan-It W. Okitsu S.. (2012). Seasonal pattern and genotype distribution of sapovirus infection in Japan, 2003-2009. Epidemiol. Infect. 140, 74–77. 10.1017/S0950268811000240 21371364

[B10] DuPont H. L. (2009). Clinical practice. bacterial diarrhea. N. Engl. J. Med. 361, 1560–1569. 10.1056/NEJMcp0904162 19828533

[B11] Dutta S. Chatterjee A. Dutta P. Rajendran K. Roy S. Pramanik K. C.. (2001). Sensitivity and performance characteristics of a direct PCR with stool samples in comparison to conventional techniques for diagnosis of *Shigella* and enteroinvasive *Escherichia coli* infection in children with acute diarrhoea in Calcutta, India. J. Med. Microbiol. 50, 667–674. 10.1099/0022-1317-50-8-667 11478669

[B12] Fischer Walker C. L. Sack D. Black R. E. (2010). Etiology of diarrhea in older children, adolescents and adults: a systematic review. PLoS Negl. Trop. Dis. 4:e768. 10.1371/journal.pntd.0000768 20689809 PMC2914743

[B13] Fukushima H. Tsunomori Y. Seki R. (2003). Duplex real-time SYBR green PCR assays for detection of 17 species of food- or waterborne pathogens in stools. J. Clin. Microbiol. 41, 5134–5146. 10.1128/JCM.41.11.5134-5146.2003 14605150 PMC262470

[B14] Harrington S. M. Buchan B. W. (2015). Multicenter evaluation of the BD max enteric bacterial panel PCR assay for rapid detection of *Salmonella* spp., *Shigella* spp., *Campylobacter* spp. (*C. jejun*i and *C. coli*), and Shiga toxin 1 and 2 genes. J. Clin. Microbiol. 53, 1639–1647. 10.1128/JCM.03480-14 25740779 PMC4400754

[B15] Iijima Y. Asako N. T. Aihara M. Hayashi K. (2004). Improvement in the detection rate of diarrhoeagenic bacteria in human stool specimens by a rapid real-time PCR assay. J. Med. Microbiol. 53, 617–622. 10.1099/jmm.0.45607-0 15184531

[B16] Khare R. Espy M. J. Cebelinski E. Boxrud D. Sloan L. M. Cunningham S. A.. (2014). Comparative evaluation of two commercial multiplex panels for detection of gastrointestinal pathogens by use of clinical stool specimens. J. Clin. Microbiol. 52, 3667–3673. 10.1128/JCM.01637-14 25100818 PMC4187753

[B17] Kotloff K. L. Nataro J. P. Blackwelder W. C. Nasrin D. Farag T. H. Panchalingam S.. (2013). Burden and aetiology of diarrhoeal disease in infants and young children in developing countries (the Global Enteric Multicenter Study, GEMS): a prospective, case-control study. Lancet 382, 209–222. 10.1016/S0140-6736(13)60844-2 23680352

[B18] Kwang J. Littledike E. T. Keen J. E. (1996). Use of the polymerase chain reaction for *Salmonella* detection. Lett. Appl. Microbiol. 22, 46–51. 10.1111/j.1472-765X.1996.tb01106.x 8588887

[B19] Letchumanan V. Yin W. F. Lee L. H. Chan K. G. (2015). Prevalence and antimicrobial susceptibility of *Vibrio parahaemolyticus* isolated from retail shrimps in Malaysia. Front. Microbiol. 6:33. 10.3389/fmicb.2015.00033 25688239 PMC4311705

[B20] Li Y. Xie X. Shi X. Lin Y. Qiu Y. Mou J.. (2014). *Vibrio parahaemolyticus*, Southern Coastal Region of China, 2007-2012. Emerging Infect. Dis. 20, 685–688. 10.3201/eid2004.130744 24655369 PMC3966377

[B21] Liu H. X. Zhang J. (2013). [Analysis of reported infectious diarrhea (other than cholera, dysentery, typhoid and paratyphoid) in China in 2011]. Zhonghua Yu Fang Yi Xue Za Zhi 47, 328–332. 10.3760/cma.j.issn.0253-9624.2013.04.009 23928638

[B22] Liu J. Kabir F. Manneh J. Lertsethtakarn P. Begum S. Gratz J.. (2014). Development and assessment of molecular diagnostic tests for 15 enteropathogens causing childhood diarrhoea: a multicentre study. Lancet Infect. Dis. 14, 716–724. 10.1016/S1473-3099(14)70808-4 25022434

[B23] Operario D. J. Houpt E. (2011). Defining the causes of diarrhea: novel approaches. Curr. Opin. Infect. Dis. 24, 464–471. 10.1097/QCO.0b013e32834aa13a 21844805 PMC3258431

[B24] Qu M. Deng Y. Zhang X. Liu G. Huang Y. Lin C.. (2012). Etiology of acute diarrhea due to enteropathogenic bacteria in Beijing, China. J. Infect. 65, 214–222. 10.1016/j.jinf.2012.04.010 22546618

[B25] Scallan E. Hoekstra R. M. Angulo F. J. Tauxe R. V. Widdowson M. A. Roy S. L.. (2011). Foodborne illness acquired in the United States–major pathogens. Emerging Infect. Dis. 17, 7–15. 10.3201/eid1701.P11101 21192848 PMC3375761

[B26] Verhoef L. Depoortere E. Boxman I. Duizer E. Van Duynhoven Y. Harris J.. (2008). Emergence of new norovirus variants on spring cruise ships and prediction of winter epidemics. Emerging Infect. Dis. 14, 238–243. 10.3201/eid1402.061567 18258116 PMC2600213

[B27] Xie X. L. Zhang W. Liao X. C. Deng X. Z. Liu L. R. Zhang Q. C.. (2012). [Molecular epidemiology of viral diarrhea in Chengdu infants and young children]. Zhonghua Shi Yan He Lin Chuang Bing Du Xue Za Zhi 26, 2–4. 10.3760/cma.j.issn.1003-9279.2012.01.002 22919739

[B28] Yu J. Jing H. Lai S. Xu W. Li M. Wu J.. (2015). Etiology of diarrhea among children under the age five in China: results from a five-year surveillance. J. Infect. 71, 19–27. 10.1016/j.jinf.2015.03.001 25753104 PMC4667737

[B29] Zhang X. E. Li D. D. Li X. Yang X. D. Cai K. Wang Y. X.. (2012). [Etiological and epidemiological study on viral diarrhea among children in Changchun]. Zhonghua Shi Yan He Lin Chuang Bing Du Xue Za Zhi 26, 5–7. 10.3760/cma.j.issn.1003-9279.2012.01.003 22919740

[B30] Zhang Y. Zhao Y. Ding K. Wang X. Chen X. Liu Y.. (2014). Analysis of bacterial pathogens causing acute diarrhea on the basis of sentinel surveillance in Shanghai, China, 2006-2011. Jpn. J. Infect. Dis. 67, 264–268. 10.7883/yoken.67.264 25056071

[B31] Zhao A. L. Xiong Y. W. Bai X. M. Zhang S. M. Wang Y. Sun H. (2011). Multiplex PCR for identification of diarrheogenic *Escherichia coli* and *Shigella* spp. Dis. Surv. 26, 65–67. 10.3784/j.issn.1003-9961.2011.01.020

[B32] Zhu M. Cui S. Lin L. Xu B. Zhao J. Xia S.. (2013). Analysis of the aetiology of diarrhoea in outpatients in 2007, Henan province, China. Epidemiol. Infect. 141, 540–548. 10.1017/S0950268812000970 22677444 PMC9152039

